# Diffusion and Adsorption of 2‑Methylpentane and 3‑Methylpentane in MFI-Type Zeolite Crystals

**DOI:** 10.1021/acs.jpcc.6c02168

**Published:** 2026-05-05

**Authors:** Patricia Seidel, Kaihang Shi, Christian Chmelik, Michael Goepel, Roger Gläser, Randall Q. Snurr, Jörg Kärger

**Affiliations:** † 9180 Leipzig University, Institute of Chemical Technology, Linnéstr. 3, Leipzig 04103, Germany; ‡ Department of Chemical and Biological Engineering, 12292 University at Buffalo, The State University of New York, Buffalo, New York 14260, United States; § 399752 Leipzig University, Faculty of Physics and Earth System Sciences, Linnéstr. 5, Leipzig 04103, Germany; ∥ 3270 Northwestern University, Department of Chemical and Biological Engineering, 2145 Sheridan Road E136, Evanston, Illinois 60208, United States

## Abstract

Due to the intimate contact of guest molecules with the inner surface of microporous adsorbents, slight changes in the structure of the guest molecules may lead to remarkable changes in their microdynamics. As an impressive example, in this work, diffusion measurements via IR microimaging (IRM) show that the transport diffusivity of 2-methylpentane (2MP) exceeds the value attained for 3-methylpentane (3MP) by a factor of 2–4.5 in silicalite-1, an MFI-type zeolite crystal and a technically relevant porous host system. This experimental finding is rationalized by comparison with the outcome of dynamically corrected transition state theory (dcTST) simulations.

## Graphical abstract

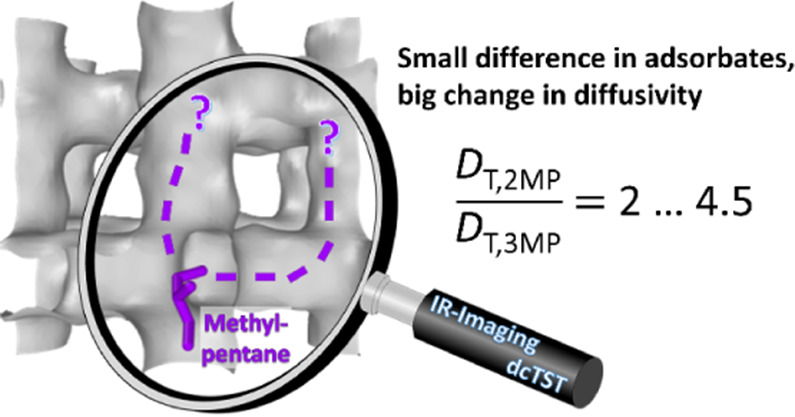

## Introduction

Microporous materials are key to numerous technological applications,^1–3^ including molecular separation, conversion and capture. In many cases, molecular diffusion within the micropores is among the intrinsic phenomena deciding the performance of these applications.^4,5^ Detailed knowledge about the rate of mass transfer is thus among the main prerequisites for an optimum design of these technological processes. Diffusion is, simultaneously, one of the fundamental, essentially omnipresent phenomena in nature.^6^ New insights may thus be of great interest for fundamental science quite in general.

Due to both their economic and scientific importance, zeolites with MFI-type structure are among the most studied zeolitic structures since several decades. A multitude of experimental^1,5^ and theoretical^7,8^ studies report on the adsorption and diffusion characteristics of a large variety of guest molecules. The crystalline structure, the intersecting pore network and the similarity in the pore sizes of the host systems with the sizes of the guest molecules have been found to lead to an impressive series of special features and particularities in their adsorption and diffusion properties. Already early works highlight features like sorbate-induced structural changes of the MFI lattice,^9,10^ stepped isotherms^11^ and preferred sorption sites^12^ and “intersection blocking”.^13^ D. N. Theodorou was among the first to apply molecular simulations to study adsorption and diffusion in zeolitic materials,^12,13^ thus pioneering the development of a new field of research which has become key for understanding and predicting adsorption and diffusion phenomena in nanoporous materials.^7,14–16^

Anomalous diffusion patterns gain particular importance if they may be used in advanced separation technologies, notably for constituents which are difficult to separate from each other by conventional distillation techniques. A prominent example is the separation of linear and branched alkanes (with different types of branching) in fuel upgrading, as a task of high economic relevance.^1,2,17,18^ Due to the similarity of their thermodynamic properties, their separation by conventional methods becomes highly cost-intensive. This makes the search for alternative processes particularly attractive.^19^ While differences in the micropore diffusivities of linear and branched alkanes can be easily rationalized already based on their different kinetic or limiting diameters, also differences in their adsorption characteristics and molecular lengths leave their imprints in the diffusion behavior, e.g., by inflections in the loading dependence,^20–23^ their packing efficiency^24,25^ or “commensurate adsorption and diffusion”.^16^ Even subtle structural differences in the guest molecule like for *p*-xylene and *o*-xylene may lead to surprisingly large differences in the intracrystalline diffusivities of 1 order of magnitude and more.^26–29^

With the present paper, we report our recent diffusion studies of two other similar molecules in silicalite-1 via IR microimaging,^30^ viz. 2-methylpentane (2MP) and 3-methylpentane (3MP). With similar molecular dimensions (see Table 1), the seemingly insignificant shift of the methyl group by a single position along the alkane chain is found to reduce the diffusion coefficient of the molecule in the MFI-type zeolite by a factor of 2–4.5, opening up the option of a diffusion-based separation of the two isomers by MFI-type membranes. Dynamically corrected transition state theory (dcTST) calculations were also performed at infinite dilution to obtain free energy profiles and estimates of the diffusion coefficients. The orientations of the molecules in the channels were also studied using Monte Carlo simulations and energy minimization method. These calculations corroborate the experimental results and provide additional atomic-level understanding.

**Table 1:** Molecular Dimensions of 2-Methylpentane (2MP) and 3-Methylpentane (3MP)

	Molecular dimensions (Å)^61^			Kinetic diameters (Å)	
	X	Y	Z	Ref 62	Ref 53
2MP	9.2	6.4	5.3	5.0	6.1
3MP	9.3	6.2	5.2	5.0	6.1

## Experimental Methods

### Synthesis and Characterization of the Material

Silicalite-1 was prepared via a synthesis adapted from Mueller and Unger^31^ by dissolving 1.42 g of tetrapropylammonium bromide (98%, Aldrich) in 15.45 g of fully deionized H_2_O. To this solution 5.91 g of Ludox AS40 (40 wt % aqueous solution, Aldrich) and then 11.15 g of NH_3_ (25 wt % aqueous solution, VWR) were added dropwise. The final synthesis mixture had a molar batch composition of 59:4:123:2280 (SiO_2_/(TPA)_2_O/(NH_4_)_2_O/H_2_O) and was transferred into a 50 cm^3^ stainless steel autoclave with a PTFE liner. The autoclave was placed into a preheated oven at 453 K and left under hydrothermal conditions for 7 days. After cooling to room temperature, the obtained silicalite-1 crystals were filtered and washed with 3× 20 mL of water before being dried for 3 h at 373 K in air. Finally, the obtained material was heated to 823 K for calcination over 10 h in air, with a heating rate for each step of 1 K/min.

The nitrogen physisorption was performed on a BELSORP-miniX (Microtrac Retsch GmbH, Germany) at 77 K. The sample was pretreated for 24 h at 523 K under vacuum before measurement.

X-ray powder diffraction investigation was carried out at room temperature using a HUBER G670-180 diffractometer (HUBER Diffraktionstechnik GmbH and Co. KG, Germany) with a measurement time of 15 min. Reflections were measured between 2θ = 4°–100° in increments of 0.005° using Cu–Kα radiation (λ = 0.154 nm).

Visual investigation and determination of crystal sizes and morphology were conducted via light microscopy, using a Motic BA210 microscope with attached camera, and Scanning Electron Microscopy was conducted with a Leo 1530 Gemini (Zeiss, Germany). For the latter, the sample was placed on a carbon conductive tab and sputtered with gold. The measurement was done using 10 kV as accelerating voltage and aperture set to 30 μm. ImageJ open source software^32^ was used to measure 100 crystals from the microscopic images and determine the average crystal dimensions.

The nitrogen adsorption isotherm (Figure S1a) confirms the microporous nature of the sample and exhibits a step between *p*/*p*_0_ = 0.1–0.2. This is due to a phase transition phenomenon of the adsorbate, characteristic for MFI-type zeolites with a high silica content.^33,34^ Investigation of the silicalite-1 sample via X-ray powder diffraction shows well-defined reflexes matching those of the MONO MFI-type framework structure for calcined crystals (Figure S1b–d).^10^ Light microscopy indicates the pyramidal segments composing the individual silicalite-1 crystals^35^ (Figure S2) and along with SEM imaging (see Figure 1c) reveals the crystals to have average dimensions of 125 × 17 × 17 μm^3^ in the typical coffin-shape of MFI framework type zeolites (Figure 1c). This makes them eminently suitable for IR microimaging studies of individual crystals. Their size is sufficient to achieve a good signal-to-noise ratio during uptake measurements, while equilibrium is reached within a measurable time frame of approximately 1 h.

**Figure 1:**
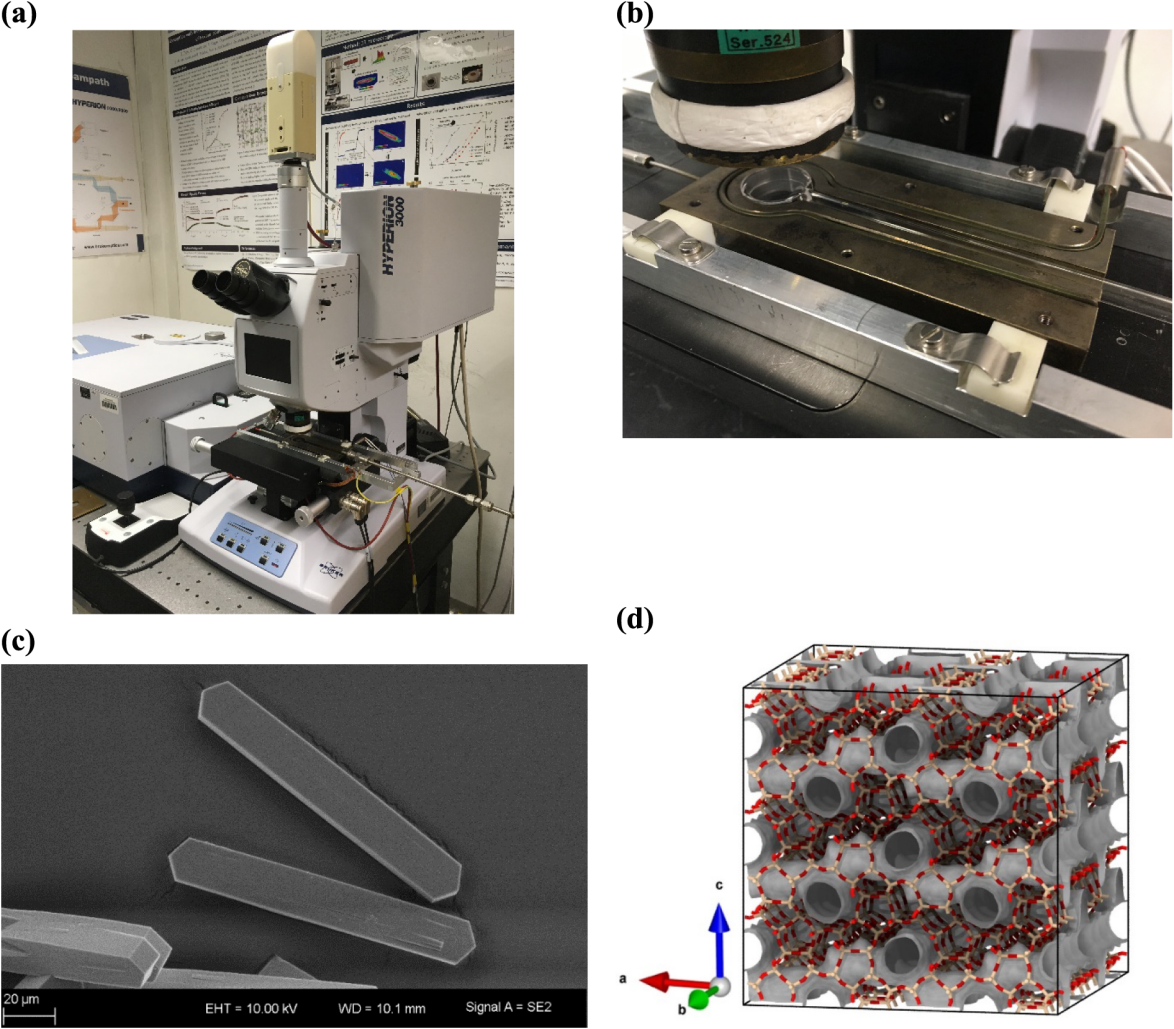
(a) IR Microscope setup with spectrometer (left) connected to the microscope and the optical cell connected to the vacuum system (out of picture, right). (b) Optical cell with a few dozen zeolite crystals. (c) SEM snapshot of the MFI-type crystals used in this study. The crystal size *l_a_* × *l_b_* × *l_c_* is approximately 17 × 17 × 125 μm^3^. (d) MONO MFI structure overlaid with its channel network (gray surface) showing the zigzag channels along the *a*-axis and the straight channels along the *b*-axis. For the framework atoms, red is oxygen and sandy brown is silicon. The figure was created using OVITO software.^37^

### IR Microimaging

Diffusion investigations were conducted with a Bruker Hyperion 3000 IR microscope attached to a Bruker Vertex 80 FT-IR spectrometer with polychromatic MIR source, Michelson Interferometer and a single element MCT (mercury cadmium telluride) detector (Figure 1a). A quartz glass optical cell containing the silicalite-1 crystals is connected to a vacuum system and the sample was activated under vacuum for 15 h at 673 K with a heating rate of 1 K/min and mounted onto the motorized stage after activation while keeping the sample under vacuum (Figure 1b). IR measurements were conducted at room temperature in transmission mode. Each uptake step was initialized by a change in the pressure of the probe molecule inside the optical cell. Probe molecules used were 2-methylpentane (99+%, Acros) and 3-methylpentane (>99%, Alfa Aesar).

Time resolved IR absorbance spectra for each pressure step were integrated, normalized, and plotted as uptake curves with absorbance over time. Apparent transport diffusivity values *D*_*T*_ were obtained from the integral uptake curves by fitting the experimental data with the solution of Fick’s second law for diffusion in a cylinder, adapted from Crank,^30,36^

1 $$A_{\mathrm{norm}}=\frac{c\left(t\right)-c_{0}}{c_{\infty }-c_{0}}=1-4\times \overset{\infty }{\underset{n=1}{\sum }}\left(\frac{1}{\alpha_{n}^{2}}\times \exp\left(-\frac{\alpha_{n}^{2}\times D_{T}\times t}{r_{cyl}^{2}}\right)\right)$$

with $J_{0}\left(\alpha_{n}^{2}\right)=0$ and *J*_0_ as the zero order Bessel function of the first kind and *t* as the time. *r_cyl_* is the radius of a cylinder with similar surface to volume ratio as the zeolite crystal (assuming cuboid shape). Then it follows *r_cyl_* = *l_a_* × *l_b_*/(*l_a_* + *l_b_*) with *l_a_* and *l_b_* as the crystal dimensions in *a*- and *b*-direction of the cross-sectional area of the cuboid crystal. Further, *c*_0_ is the concentration at *t* = 0, *c*(*t*) the mean concentration at a given time *t,* and *c*_∞_ the equilibrium concentration after the adsorption step. Examples of uptake curves and fits are included in the Supporting Information (see Figures S4–S6).

## Simulations

### Molecular Simulation of Adsorption

Grand canonical Monte Carlo (GCMC) simulations were carried out using the RASPA2 package^38,39^ to predict the single-component adsorption isotherms of 2MP and 3MP in MONO MFI^40^ at 300 K. The MFI structure was treated as rigid during the simulations. We note that while accounting for the flexibility of MFI is important for accurate modeling of the adsorption and diffusion of bulky aromatic molecules like xylene,^41^ the adsorptive behavior of linear and branched alkanes such as 2MP and 3MP have been shown to be less affected by the flexibility of the MFI structure.^42,43^ The good agreement between simulation and experimental results in this study further supports the rigid approximation of the MFI structure (see Results and Discussion section). The influence of structural flexibility on diffusion of alkanes will be the subject of future studies. Force field parameters for both adsorbate–adsorbate and adsorbate–zeolite interactions were taken from Dubbeldam et al.^43^ They generated the force field by adjusting the parameters to faithfully reproduce the experimental isotherms of both linear and branched alkanes in MFI-type zeolite over a wide range of pressures and temperatures. In particular, this force field reproduces Henry’s constants very well for linear and branched alkanes (including 2MP and 3MP) in (ORTHO) MFI, although validation against experimental data was only possible at an elevated temperature. Indeed, our dcTST calculations below confirm the validity of the force field at infinite dilution condition. Nonbonded interactions were modeled by the standard 12–6 Lennard-Jones (LJ) potential. Each alkane molecule was simulated as a flexible united atom model. Beads were connected by a harmonic potential. Angle bending and torsion were controlled by a harmonic cosine bending potential and a Ryckaert-Bellemans potential, respectively. Force field parameters are tabulated in Tables S2–S5. All nonbonded interactions were shifted and cut at 12.0 Å without tail corrections. The MFI unit cell was replicated to create a 2 × 2 × 2 supercell to ensure that the distance between two opposing cell surfaces is at least twice the cutoff distance. The number of initialization and production cycles were both set to 5 × 10^5^, where a cycle consists of *N* steps, where *N* is the number of molecules, with a minimum of 20 steps. The Monte Carlo moves used were translation, rotation, partial and full reinsertion of the molecule, and swap moves (insertions and deletions), and these moves were attempted with equal probability. For reinsertion and swap moves, the configurational bias algorithm^44^ was implemented to enhance the sampling of molecular configurations. RASPA simulation input files and MFI CIF files are available in the Supporting Information.

### Dynamically Corrected Transition State Theory Calculations

Slow diffusion with self-diffusivities below about 10^–12^ m^2^/s is inaccessible to the (currently achievable) time scale of molecular dynamics (MD) simulations. To estimate the self-diffusivities of 2MP and 3MP in MONO MFI, we turned to the dcTST method.^45^ We consider a system having two stable states, A and B. The reaction coordinate, *q*, depicts the progress of the diffusion event from state A (*q_A_*) to state B (*q_B_*). In a free energy profile, *F*(*q*), along the reaction coordinate, these two stable states are minima separated by a dividing surface located at q*. By the symmetry of the MFI channels, the reaction coordinate *q* was defined as^46^

2 $$q=s_{a}\left(\mathrm{for}\;\mathrm{diffusion}\;\mathrm{in}\;\mathrm{the}\;\mathrm{zigzag}\;\mathrm{channel}\right)$$

3 $$q=s_{b}\left(\mathrm{for}\;\mathrm{diffusion}\;\mathrm{in}\;\mathrm{the}\;\mathrm{straight}\;\mathrm{channel}\right)$$

where *s_a_* is the fractional coordinate (in the unit cell) along the *a*-axis and *s_b_* is the fractional coordinates along the *b*-axis. We chose the fractional coordinates to be the location of the branched bead in 2MP and 3MP because this choice leads to the highest free energy barrier and a fast converging transmission coefficient.^47^

In the Bennett-Chandler approach,^45,48^ the hopping rate from state A to state B over the barrier q* can be calculated by^49^

4 $$k_{AB}=\kappa k_{AB}^{TST}$$

where κ is the transmission coefficient. Transition state theory (TST) assumes that all systems that reach the barrier will equilibrate in the final state. In reality, however, it is possible that a trajectory crosses the barrier but fails to end up in the final state (*i.e*., recrossing the barrier) because of, for example, the orientation of the tail in a branched alkane.^50^ The transmission coefficient is a dynamic correction factor to account for these situations. The parameter $k_{AB}^{TST}$ in eq 4 is the TST hopping rate,

5 $$k_{AB}^{TST}=\sqrt{\frac{k_{B}T}{2\pi m}}\frac{e^{-\beta F\left(q^{*}\right)}}{\int_{\mathrm{region}A}e^{-\beta F\left(q\right)}dq}$$

where $\sqrt{k_{B}T/2\pi m}$ is half of the average velocity drawn from the Maxwell–Boltzmann distribution in a one-dimensional system; *k*_B_ is the Boltzmann constant, *T* is the system temperature, and *m* is the mass of the particle involved in the reaction coordinate (in our case, it is the mass of the [CH] bead that carries the branched methyl group). The term to the right of $\sqrt{k_{B}T/2\pi m}$ in eq 5 is the probability of finding the system on the dividing surface relative to the probability of finding it in state A, and $\beta =\frac{1}{k_{B}T}$ .

The calculation of the hopping rate defined in eq 4 involved two steps. We first calculated the excess free energy profile using the Widom insertion method,^51^

6 $$\beta F_{ex}\left(q\right)=-\ln\left\langle e^{-\beta \Delta V}\right\rangle$$

where ΔV is the potential energy of the probe molecule with the host structure; $\left\langle e^{-\beta \Delta V}\right\rangle$ was calculated as the ensemble average by placing the branched bead on the hyperplane perpendicular to the reaction coordinate, and all other degrees of freedom were then sampled. The Widom insertion moves were performed at least 8 × 10^7^ times in an empty framework structure (*i.e*., at infinite dilution) to achieve a reliable free energy profile. For the calculation of the probability density of finding the system on the dividing surface in eq 5, the raw free energy profile was fitted to a cubic spline curve (Figure S8) for the convenience of the numerical integration.

Next, the transmission coefficient κ was calculated from short microcanonical (NVE) molecular dynamics (MD) simulations with a time step of 0.5 fs. A separate canonical MC simulation was performed to generate at least 30,000 single-molecule configurations as the initial configurations for the MD simulations. These single-molecule configurations were thermalized in the MC simulations with the branched bead in 2MP and 3MP constrained on the dividing surface (chosen to be at top of the barrier q* here). Initial velocities for NVE-MD simulations were drawn from the Maxwell–Boltzmann distribution at 300 K, with equal numbers of trajectories directed forward and backward. The reported κ is the converged value after a 10 ps simulation, calculated as the net fraction of trajectories that thermalized in the final state.^52^ If every trajectory crossed the dividing surface and equilibrated in the final state, the transmission coefficient would be κ = 1. More details on transmission coefficient calculations are available in SI. All dcTST and (excess) free energy calculations were performed at 300 K in the MONO MFI structure using the RASPA2 package.^38,39^ Relevant simulation files are available in the SI, as well as a short consideration on the choice of the MONO MFI structure (see Page S4).

## Results and Discussion

To set the background for studying the diffusion behavior, adsorption isotherms were recorded for 2MP and 3MP using IR microimaging (see Figure 2). The relative loadings obtained in the IR measurements were normalized by using the inflection point as reference for a loading of 4 molecules per unit cell (molec/uc). For both molecules a step in the isotherm is expected at this loading, since the intersections act as preferred adsorption sites for branched alkanes, as already pointed out by Theodorou and coworkers back in 1990.^12^ Saturating all 4 intersections available per unit cell leads to a more or less pronounced plateau as the adsorption strength in the “less comfortable” channel segments is significantly smaller. While this explanation is generally accepted for more than 30 years now, a quantitative comparison of 2MP and 3MP adsorption is still challenging.

**Figure 2:**
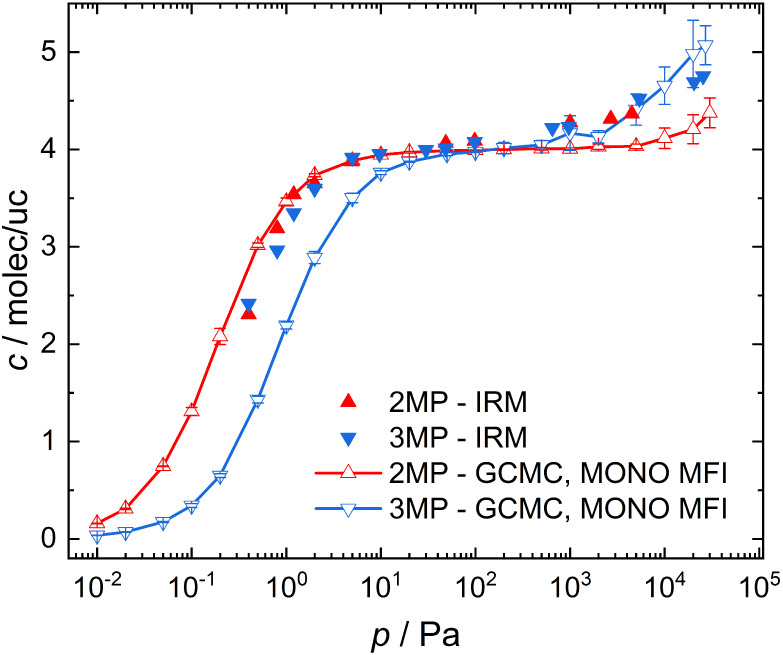
Comparison of the IRM adsorption isotherms of the methyl-pentane isomers 2MP and 3MP in MONO MFI zeolites at 300 K with results from GCMC simulations.

Experimental studies of 2MP and 3MP in MFI-type zeolites in the literature revealed rather similar sorption properties for both molecules. Although the characteristic parameters like isosteric heat of adsorption and Henry’s constant show deviations between different reports near or within the range of the experimental uncertainty, two features seem to be reproducible: while 2MP adsorbs somewhat stronger at loadings <4 molec/uc and its equilibrium loading is surpassed by 3MP in the range >4 molec/uc (see Figure S3 and refs 53, 54 ). To the best of our knowledge, experimental results on the low-loading range were reported only for temperatures of 373 K and above. In our IRM studies at 300 K the adsorption data of both molecules essentially coincide within the experimental uncertainty, even though indications for both features can be recognized.

For loadings above 4 molec/uc also a guest-induced reversible change of the MFI structure from MONO to ORTHO is expected (see also Page S4 in the Supporting Information).^10,55–58^ As the experimental part of the study was done in a loading range below the transition loading, our simulations were focused on the MONO MFI structure.

GCMC predictions for 2MP and 3MP adsorption in MONO MFI structure^40^ using force field parameters from Dubbeldam et al. (see Tables S2–S5)^43^ are included in Figure 2. Simulation data for ORTHO MFI^59^ and PARA MFI^10^ structures are available in Figure S3a. While simulation results generally agree with the IRM data, simulation predicted a more pronounced difference in adsorption loading between 2MP and 3MP than observed in experiment. At loadings <4 molec/uc, simulation predicted larger adsorption loading of 2MP compared to that of 3MP, consistent with the higher isosteric heat of 2MP under this condition (Figure S7). At loadings >4 molec/uc, the trend reverses, and 3MP exhibits higher simulated adsorption amount than 2MP, consistent with the reversed behavior of isosteric heat beyond 4 molec/uc (Figure S7). The higher adsorption amount for 2MP than for 3MP was also observed in GCMC adsorption isotherm at a higher temperature, 373 K (Figure S3c). The discrepancies between the simulation and experiments may be due to limitations of the force field. We also carried out GCMC simulations using the TraPPE-zeo force field.^60^ Unlike force field parameters from Dubbeldam et al.^43^ that are tailored for alkanes in MFI-type zeolites, the TraPPE-zeo force field is calibrated using adsorption data of diverse molecules (e.g., *n*-heptane, carbon dioxide) in MFI- and TON-type zeolites, aimed at being transferable for a wide variety of molecules in all-silica zeolites. TraPPE-zeo’s transferability has been demonstrated by good agreement between simulated and experimental adsorption isotherms for 2MP and 3MP in ORTHO MFI zeolites.^60^ Comparison of the current force field with the TraPPE-zeo force field shows good agreement of both 2MP and 3MP isotherms at loadings >4 molec/uc (Figure S3b–c). TraPPE-zeo force field also predicts higher adsorption amount for 2MP than 3MP at loadings <4 molec/uc but with the isotherms shifted toward higher pressures (Figure S3b–c).

After these considerations on the adsorption trends, we come to the main finding of this paper: despite the similar molecular dimensions of 2MP and 3MP^53^ (see Table 1) and similar sorption isotherms, the transport diffusivity of 2MP is found to be faster than that of 3MP for all considered loadings, specifically by a factor of about 2 at low loadings and increasing to about 4.5 at a loading of 4 molec/uc as shown in Figure 3.

**Figure 3:**
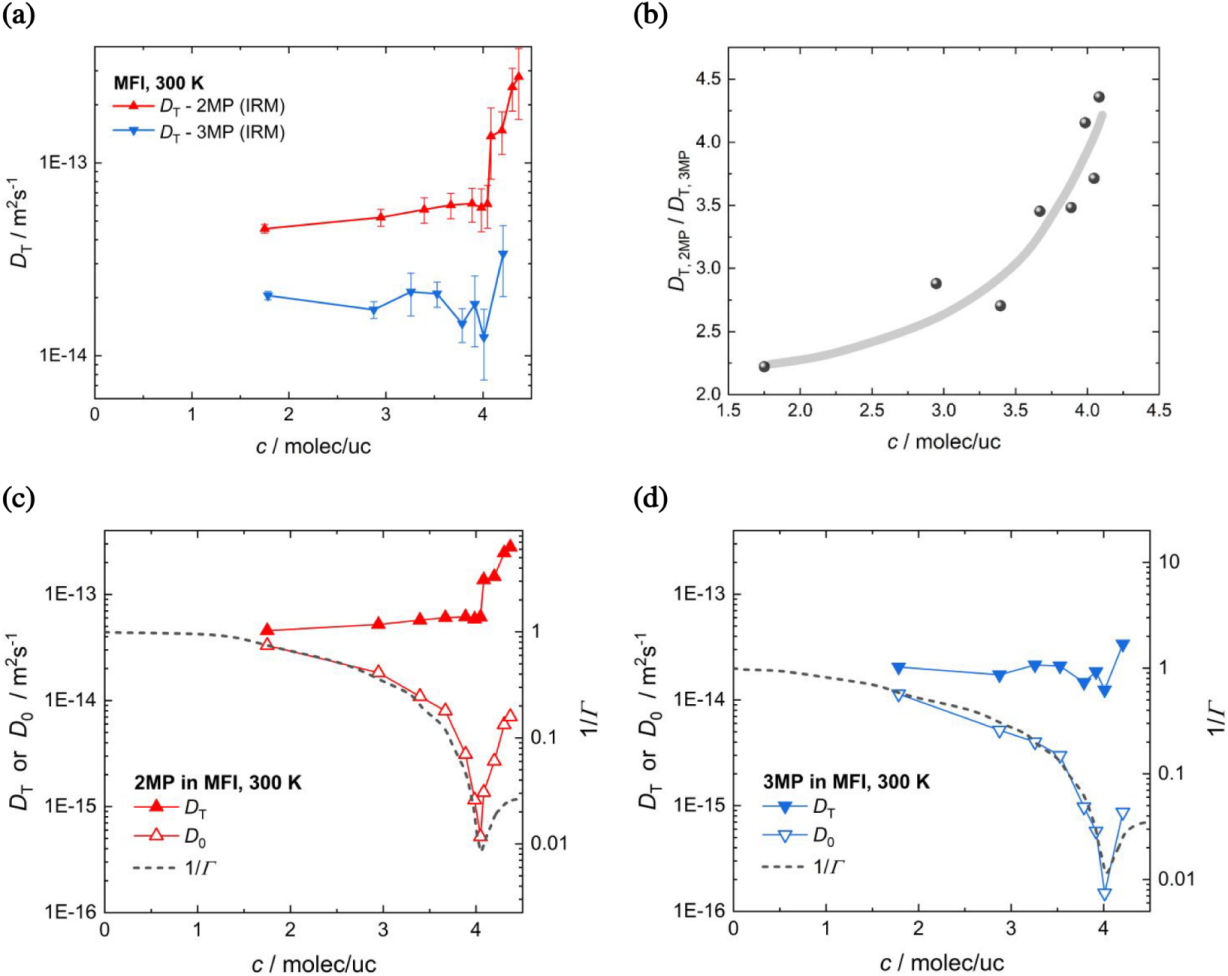
(a) Transport diffusivities of the methyl-pentane isomers 2MP and 3MP in MFI-type zeolites at 300 K from experimental measurement via IRM. (b) Ratio of the transport diffusivities, *D*_T,2MP_/*D*_T,3MP_ as a function of the guest loading. (c,d) Transport diffusivities (*D*_T_) together with the corrected diffusivities *D*_0_ ≡ *D_T_*/Γ with Γ ≡ ∂ ln *p*/∂ ln *c* denoting the “thermodynamic factor” from the adsorption isotherm *c*(*p*).

The absolute values of the diffusivities are in the range of previously reported data. While more than an order of magnitude larger than the early data of Prinz and Riekert^63^ (ca. 10^–15^ m^2^ s^–1^ for 3MP at 296 K), there is good agreement with the measurements of Millot et al.^64^ (6.5 × 10^–14^ m^2^ s^–1^ for 3MP at 303 K), Cavalcante and Ruthven^65^ (3.5 × 10^–13^ m^2^ s^–1^ and 1.3 × 10^–13^ m^2^ s^–1^ for 2MP and 3MP at 373 K, respectively, already indicating a similar ratio), Xiao and Wei^66^ (1.2 × 10^–14^ m^2^ s^–1^ for 3MP at 296 K) and Kulkarni and Anthony (1.2 × 10^–14^ m^2^ s^–1^ for 3MP at 303 K).^65^

To the best of our knowledge there has been no study which compares the mobility of these two molecules at room temperature. The loading dependencies of the transport diffusivities follow the expected trend: constant or mildly increasing diffusivity in the loading range below 4 molec/uc, but strongly increasing for loadings >4 molec/uc. The remarkable change in the loading dependencies above 4 molec/uc can be attributed to the population of “uncomfortable” channel sites, leading to a push on the molecules sitting at intersection sites and, thus, increasing the mobility of 2MP or 3MP.

Another consequence of the more or less constant transport diffusivity at loadings <4 molec/uc is that the loading dependence of the corrected diffusivity (which is equal to the Maxwell–Stefan (M-S) diffusivity for single components) follows the trend dictated by the thermodynamic factor Γ (see Figure 3c and d). This can be directly seen when comparing the Fickian and the Maxwell–Stefan approaches, yielding the relation often referred to as the Darken equation:^5^

7 $$D_{T}=D_{0}\frac{\partial \;\ln p}{\partial \;\ln c}=D_{0}\times \Gamma$$

where *D*_0_ is the corrected diffusivity, *p* is pressure and *c* is adsorbed concentration. A simple picture for the trend of 1/Γ follows from the M-S model, where it can be understood as available vacancy.^7^ Hence, a minimum is expected at a loading of 4 molec/uc, where all preferred sites are occupied. Similar trends have been observed before for a number of other guest molecules, in particular for single-branched alkanes.^20,21,67^

A variety of potential reasons could explain the difference in the mobilities of 2MP and 3MP in MFI-type structures, e.g., lower energy barriers for jumps of 2MP due to the longer “tail” sticking into channel segments, differences in the energy profiles in general or in the orientation of the two molecules which facilitate jumps of 2MP.^65^ Molecular simulations were carried out to aid the interpretation of this finding.

Figure 4 shows the free energy profiles for 2MP and 3MP in the zigzag and straight channels. States 1 and 4 correspond to the stable regions at the intersections. These two stable states are separated by two local minima, states 2 and 3, inside the channels. The free energy barrier separating states 1 and 2 is the highest among all barriers along both channel systems. Comparing 2MP and 3MP, the free energy profiles are similar for the two molecules in both types of channels. Differences in the free energy profile that contribute to the difference in the self-diffusivity of the two molecules will be discussed later.

**Figure 4:**
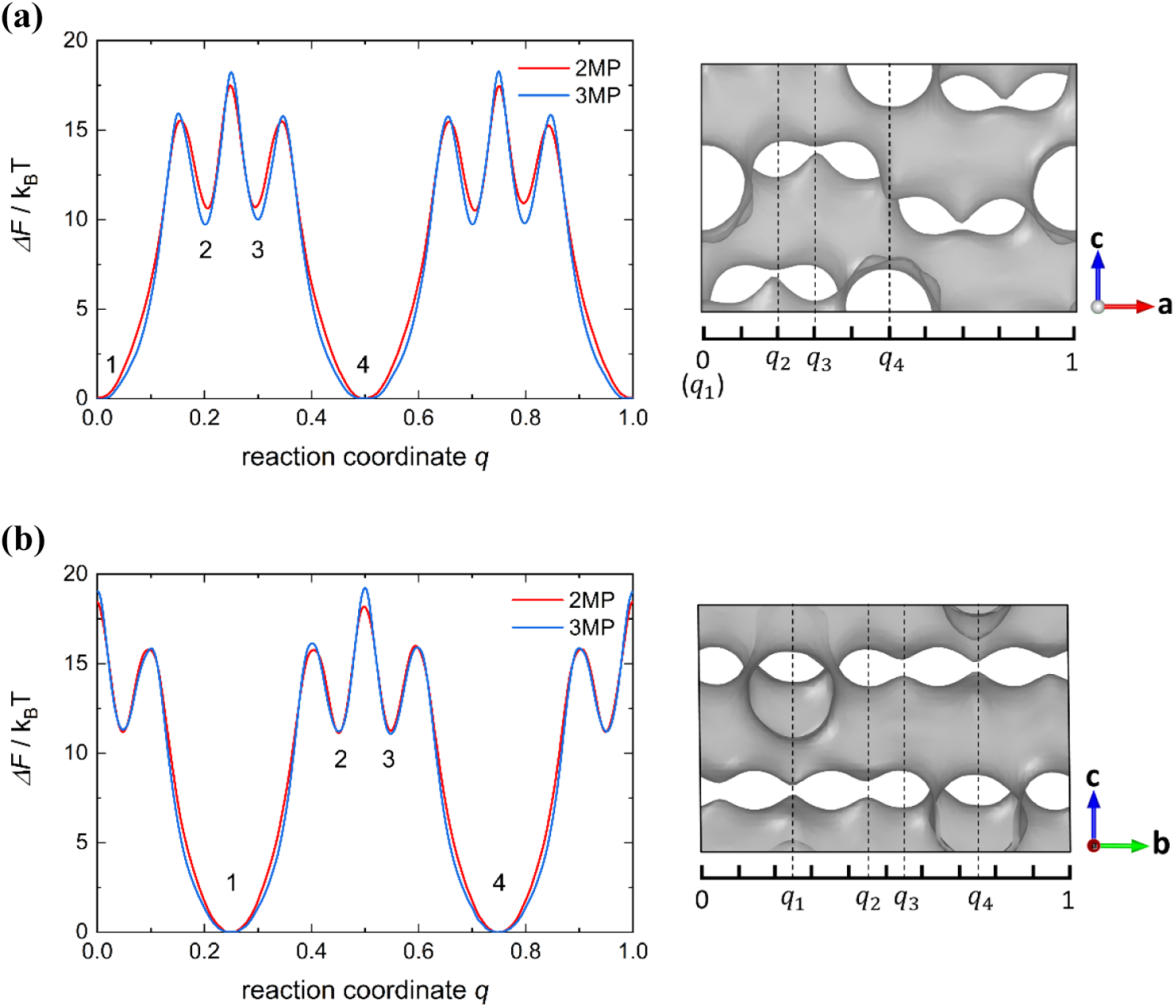
Free energy profile Δ*F*(*q*) along the (a) zigzag channel (along *a*-axis) and (b) straight channel (along *b*-axis) for 2MP and 3MP in a unit cell of the MONO MFI structure. Fitted free energy profiles using cubic splines are shown here for clarity, see Figure S9 for the original data. Free energy profiles are shifted so that the global minimum is 0. Corresponding free energy local and global minima are marked in both free energy profiles (left, as 1, 2, 3, 4) and in the MFI structure (right, as corresponding *q*_1_, *q*_2_, *q*_3_, *q*_4_). In the images on the right, only the channels of MFI are shown (gray surface) for clarity, which were generated using OVITO software.^37^

To connect the free energy profiles to the diffusion coefficients, we used dcTST calculations to obtain the self-diffusivities at infinite dilution, noting that the self-, transport and corrected diffusivities coincide at zero loading and can be directly compared.^5^ If we consider the hopping event in both zigzag and straight channels as 1D diffusion, the self-diffusivity at infinite dilution in both channels can be calculated by^49,50,68^

8 $$D_{s,\mathrm{zigzag}}=\frac{1}{4}k_{14,zz}a^{2}$$

9 $$D_{s,\mathrm{straight}}=\frac{1}{4}k_{14,st}b^{2}$$

where parameters *a* and *b* are the unit cell lattice parameters. Hopping rate constants *k*_14,*zz*_ and *k*_14,*st*_ are those between intersections (from state 1 to state 4 in Figure 4) in the zigzag (*zz*) and straight (*st*) channels, respectively. Here, we estimated the hopping rate constant *k*_14_ using two different free energy barriers: (1) the *local* free energy barrier separating states 1 and 2 (e.g., q* ≈ 0.15 in zigzag channel in Figure 4a), which is much higher than the other local barriers and can be considered as the rate-limiting step,^68^ and (2) the *global* free energy barrier, suggested by the energetic span model^69^ to be the highest energy barrier between states 1 and 4 (e.g., q* ≈ 0.25 in zigzag channel in Figure 4a). With the local barrier assumption, $k_{14}\approx \kappa_{12}k_{12}^{TST}$ , where $k_{12}^{TST}$ is the TST hopping rate from states 1 to 2. With the global barrier assumption, $k_{14}\approx \kappa_{12}k_{14}^{TST}$ , where $k_{14}^{TST}$ is the effective, single-step hopping rate from states 1 to 4 determined by the free energy difference between the global energy maximum and global minimum. The same transmission coefficient from states 1 to 2 was used in both cases for convenience. By considering the diffusion in *c*-direction as a two-step hopping process: from intersection (state) A to intersection B through the straight channel, and then from B to intersection C through the zigzag channel, we can then calculate the hopping rate constant by^68,70,71^

10 $$k_{c}=\frac{k_{14,zz}k_{14,st}}{k_{14,zz}+k_{14,st}}$$

And the self-diffusivity in *c*-direction is given by^68^

11 $$D_{s,c}=\frac{1}{4}k_{c}c^{2}$$

The overall (directionally averaged) self-diffusion coefficient is

12 $$D_{s,ave}=\frac{D_{s,zigzag}+D_{s,straight}+D_{s,c}}{3}$$

Hopping rate constants, transmission coefficients, and self-diffusivities in the zigzag and straight channels as well as in the *c*-direction are presented in Table 2. In general, the estimated self-diffusivities for both molecules follow the ordering: zigzag > straight > *c*-direction. The higher self-diffusivity in the zigzag channel compared to the straight channel results from a larger TST hopping rate constant $k_{12}^{TST}$ (using local free energy barrier) or $k_{14}^{TST}$ (using global free energy barrier), and a larger transmission coefficient. A larger transmission coefficient in the zigzag channel compared to that in the straight channel suggests that the transport in the zigzag channel is less affected by the orientation (of the “tail”) of the molecule located at the dividing surface.

**Table 2:** Results of the dcTST Calculations for the Self-Diffusion of 2MP and 3MP in the MONO MFI Structure at Infinite Dilution and 300 K

	2MP			3MP		
	Zigzag	Straight	*c*-direction	Zigzag	Straight	*c*-direction
$k_{12}^{TST}$ [1/s]^a^	3.10 × 10^5^	1.91 × 10^5^		1.77 × 10^5^	1.11 × 10^5^	
$k_{14}^{TST}$ [1/s]^a^	3.28 × 10^4^	1.67 × 10^4^		1.18 × 10^4^	5.03 × 10^3^	
κ_12_ ^b^	0.252	0.168		0.212	0.174	
*k*_14_ [1/s] (Local/Global)^c^	7.81 × 10^4^/8.28 × 10^3^	3.22 × 10^4^/2.80 × 10^3^		3.75 × 10^4^/2.50 × 10^3^	1.94 × 10^4^/8.75 × 10^2^	
*D_s_* [m^2^/s] (Local/Global)^d^	7.90 × 10^–14^/8.37 × 10^–15^	3.18 × 10^–14^/2.77 × 10^–15^	1.02 × 10^–14^/9.35 × 10^–16^	3.79 × 10^–14^/2.52 × 10^–15^	1.92 × 10^–14^/8.64 × 10^–16^	5.70 × 10^–15^/2.89 × 10^–16^
*D*_*s*,*ave*_ [m^2^/s] (Local/Global)^e^	4.03 × 10^–14^/4.02 × 10^–15^			2.09 × 10^–14^/1.23 × 10^–15^		

a Using eq 5. See text for details.

b The standard deviation of converged κ is generally on the order of 10^–5^ (see Table S6) which is negligible compared to the precision of the reported values (3 significant figures). Therefore, uncertainties are not reported here.

c Using eq 4. See text for details about the local and global barrier assumptions.

d Using eqs 8, 9 and 11. For the calculation of *D_s_*,*_c_*, eq 10 was used to estimate the hopping rate in *c*-direction.

e Using eq 12.

Comparing 2MP and 3MP, the predicted self-diffusivity of 2MP is larger than that of 3MP in both types of channels within each respective barrier assumption (local or global), with an enhancement by a factor of 1.7–3.3. The enhancement of diffusion for 2MP can be mainly attributed to the difference in the free energy profiles in Figure 4. While 2MP and 3MP have similar energy barriers, 3MP shows a slightly wider free energy valley compared to that of 2MP. A wider free energy valley leads to a smaller TST hopping rate $k_{12}^{TST}$ or $k_{14}^{TST}$ given the same free energy barrier (eq 5), as the molecule has a higher probability to stay in the stable region than climb up the barrier. More interestingly, while several simplifications were introduced in the calculations, the overall self-diffusivities *D_s_*,*_ave_* estimated by the dcTST method for 2MP (4.03 × 10^–14^ m^2^/s with the local barrier and 4.02 × 10^–15^ m^2^/s with the global barrier) and 3MP (2.09 × 10^–14^ m^2^/s with the local barrier and 1.23 × 10^–15^ m^2^/s with the global barrier) are in qualitative agreement with previous experimental studies (e.g., for 3MP Prinz and Riekert^63^ (ca. 10^–15^ m^2^ s^–1^ at 296 K), Millot et al.^64^ (6.5 × 10^–14^ m^2^/s at 303 K), Xiao and Wei^66^ (1.2 × 10^–14^ m^2^ s^–1^ at 296 K) and Kulkarni and Anthony^65^ (1.2 × 10^–14^ m^2^ s^–1^ for 296 K) and the IRM measurements in Figure 3 (∼4 × 10^–14^ m^2^/s for 2MP and ∼2 × 10^–14^ m^2^/s for 3MP by extrapolation to loading of 1 molecule/uc). Such agreement further supports the possibility to separate 2MP and 3MP in MFI by diffusion.

When comparing the results of simulations with those of experiments, one has to bear in mind the internal twinning of MFI-type crystals.^35,72^ With an orientation of the *b*-axis (straight channels) of the pyramidal segments parallel to the outer surface and the *a*-axis (zigzag channels) perpendicular to it,^35^ one may argue that transport in the crystals used here is dictated by the zigzag channels. However, for branched alkanes in similar MFI-type crystals no difference between transport along both crystal axes could be evidenced.^22^ Hence, we conclude that such detailed consideration is beyond the level relevant for the length scale of the IR experiments and stick to our direct comparison of the averaged self-diffusivity from dcTST and the transport diffusivity calculated from the IR data.

To gain molecular insights into the enhanced diffusion for 2MP over 3MP, we analyzed the tail orientation of 2MP and 3MP molecules in the channel intersections. We defined a tail orientation order parameter, sin­(ϕ). The angle ϕ is defined between vectors **b** and **m**, where *b* is the unit lattice vector in the direction parallel to the straight channel and **m** is the molecular tail vector, defined from the branched bead to the end bead in the long tail part of the carbon backbone (see Figure S10). We collected 50,000 single-molecule configurations from a short NVT-MC simulation at 300 K with a 2MP or 3MP molecule initially placed at the channel intersection. Figure 5a shows the probability density distribution of the tail orientation order parameter sin­(ϕ) for 2MP and 3MP. For 2MP, a bimodal distribution is observed, indicating two distinct tail orientations of 2MP at the intersection. The first peak near sin­(ϕ) ∼ 0.3 corresponds to molecular configurations with the tail pointing in the direction of the straight channel. The reason for the shift of the peak location from sin­(ϕ) = 0 is that the stable orientation of the tail vector **m** is not exactly aligned parallel to the unit lattice vector **b**. The second peak at sin­(ϕ) = 1 belongs to configurations with the tail pointing toward the zigzag channel. The peak mean at exactly sin­(ϕ) = 1 can be understood by the orthogonal arrangement of the zigzag and straight channels. In contrast, the probability density distribution of the tail orientation for 3MP is unimodal with a similar peak at sin­(ϕ) = 1 and a “shoulder” extending toward lower sin­(ϕ) range. This unimodal distribution suggests that 3MP can adjust itself more freely than 2MP at the intersection with more intermediate tail orientations between the straight and zigzag channels.

**Figure 5:**
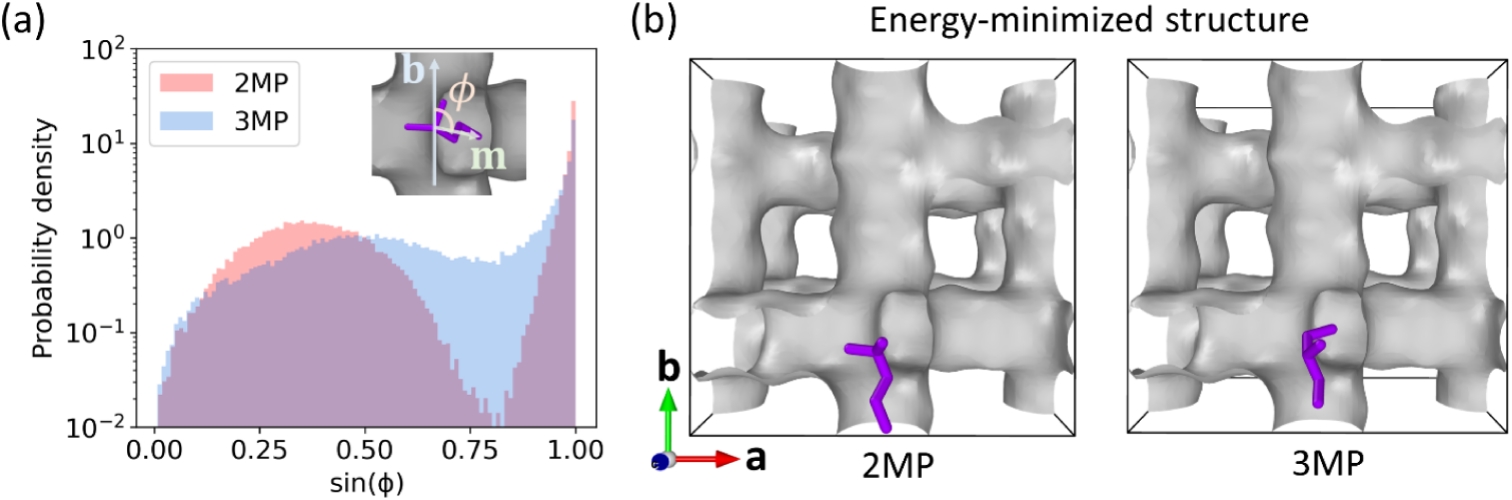
(a) Probability density distribution of the tail orientation order parameter sin­(ϕ) for 2MP and 3MP at the channel intersection of the MONO MFI structure at 300 K. The inset illustrates the definition of the angle ϕ between lattice vector **b** and molecular tail vector **m**. (b) Snapshots showing the energy-minimized molecular structure for 2MP and 3MP at the channel intersection at 0 K. Only channels (gray surface) of MONO MFI are shown for clarity. Molecules are shown in purple.

Further insights in this regard can be obtained from energy-minimized molecular structures at the intersection. We generated 500 initial random configurations for 2MP and 3MP sitting at the intersection. Baker’s algorithm^73^ implemented in the RASPA2 package^38,39^ was then performed on these 500 configurations to find the energy minima. All minimizations were finished within 100 iterations with the stopping criterion of root-mean-square gradient of 1 × 10^–6^ K/Å. The structures with minimum energy among these 500 candidates are shown in Figure 5b for 2MP and 3MP. The snapshot shows that 2MP has a favorable tail orientation toward the straight channel, and this is consistent with Figure 5a. By integrating the probability density distribution of each peak in Figure 5a, we find almost equal tendency for 2MP to relax its tail toward either the straight channel (51%) or the zigzag channel (49%), with a slight preference for the straight channel. We note that the energy-minimized structure is at 0 K and the tail orientations were sampled at 300 K. In comparison, 3MP has one of its short tails oriented toward the straight channel and the other pointed to the zigzag channel.

Combining the information from free energy profiles, dcTST calculations, and distributions of the tail orientation, the underlying mechanism for the enhanced self-diffusivity of 2MP over 3MP is as follows. For 2MP, the clear orientation of the long tail toward either the zigzag or straight channel, as reflected by the narrower free energy valley, facilitates the hopping of the molecule over the energy barrier to the neighboring intersections. In contrast, 3MP can comfortably sit inside the intersection with more free orientation of its short tail. The wider free energy valley compared to that of 2MP contributes to the propensity for 3MP to stay at the intersection rather than to jump over the barrier.

## Conclusions

Diffusion and adsorption of 2-methylpentane (2MP) and 3-methylpentane (3MP) in MFI-type zeolite crystals has been investigated using IR microscopy (IRM), grand canonical Monte Carlo (GCMC) and dynamically corrected transition state theory (dcTST) simulations. For both molecules the expected step in the isotherm at a loading of 4 molec/uc was found, since the intersections of MFI act as preferred adsorption sites for branched alkanes. Saturation of all 4 intersections available per unit cell leads to a plateau, as the adsorption strength in the “less comfortable” channel segments is significantly smaller. While this finding has been rationalized in molecular simulations more than 30 years ago,^12^ we find that isotherm predictions using molecular simulations are quite sensitive to the choice of force field and MFI-structure. Experimental studies revealed rather similar sorption properties for both molecules. Although the characteristic parameters like isosteric heat of adsorption and Henry’s constant in the literature show deviations near or within the range of the experimental uncertainty, two features seem to be reproducible and are in agreement with molecular simulations: while 2MP adsorbs somewhat stronger at loadings <4 molec/uc, its equilibrium loading is surpassed by 3MP in the range >4 molec/uc. In our IRM studies at 300 K the adsorption data of both molecules essentially coincide within the experimental uncertainty, even though indications for both features can be recognized.

The transport diffusivity of 2MP was found to exceed that of 3MP by a factor of 2–4.5, which increases with loading, despite the similar sizes of the two molecules. Molecular simulations that include free energy profiles, dcTST calculations and distributions of the alkane tail orientations provided atomic-level insights that explain the importance of a seemingly insignificant shift of the methyl group by a single position along the alkane chain for diffusion. For 2MP, the clear orientation of the long tail toward either the zigzag or straight channel facilitates the hopping of the molecule over the energy barrier to the neighboring intersections. In contrast, 3MP can comfortably sit inside the intersection with more free orientation of its short tails. A wider free energy valley compared to that of 2MP contributes to the propensity for 3MP to stay at the intersection rather than to jump over the barrier. Moreover, using the rigid MONO MFI-structure, commonly accepted force field parameters, and simplified free energy barrier assumptions, the diffusivities of both molecules obtained in dcTST calculations are in qualitative agreement with the experimental data. For the most rigorous approach, however, we recommend calculating hopping rates with framework flexibility and employing kinetic Monte Carlo simulations to obtain the diffusivities from the hopping rates.

## Supplementary Material





## Notes

Details about materials characterization; IR microimaging data analysis; and additional simulation details and results (PDF)
Simulation input files for GCMC; dcTST; Baker minimization calculations (ZIP)

## Abbreviations

2MP: 2-methylpentane
3MP: 3-methylpentane
dcTST: dynamically corrected transition state theory
GCMC: grand canonical Monte Carlo
IRM: infrared microscopy
molec/uc: molecules per unit cell
MONO MFI: monoclinic MFI framework type (*P*2_1_/*n*.1.1 symmetry)
NVT-MC: Monte Carlo simulations using the canonical ensemble (*N*, *V*, *T*)
ORTHO MFI: orthorhombic MFI framework type (*Pnma* symmetry)
PARA MFI: orthorhombic MFI framework type (*P*2_1_2_1_2_1_ symmetry)
