# Supplementary material for: Diffusion and Adsorption of 2‑Methylpentane and 3‑Methylpentane in MFI-Type Zeolite Crystals
Source: J Phys Chem C Nanomater Interfaces. 2026 May 5;130(20):7242–52. doi: 10.1021/acs.jpcc.6c02168 (PMC13200187; doi:10.1021/acs.jpcc.6c02168)
Supplement: Supplementary file 1 [file jp6c02168_si_001.pdf]

Supporting Information

Diffusion and Adsorption of 2-Methylpentane and 3-Methylpentane in MFI-Type Zeolite Crystals

Patricia Seidel[1‡], Kaihang Shi[2,4‡], Christian Chmelik[3,], Michael Goepel[1], Roger Gläser[1], Randall Q. Snurr[4], Jörg Kärger[3]*

[1] Leipzig University, Institute of Chemical Technology, Linnéstr. 3, 04103 Leipzig, Germany

[2] Department of Chemical and Biological Engineering, University at Buffalo, The State University of New York, Buffalo, New York 14260, United States

[3] Leipzig University, Faculty of Physics and Earth System Sciences, Linnéstr. 5, 04103 Leipzig, Germany

[4] Northwestern University, Department of Chemical and Biological Engineering, 2145 Sheridan Road E136, Evanston, IL 60208, USA

Contents

Section SI1. Characterization of silicalite-1 sample 3
Section SI2. IR, simulation, and literature data 7
Section SI3. IR micro-imaging data processing 9
Section SI4. Details for grand canonical Monte Carlo simulations and transition state theory calculations 11
References 15

Section SI1. Characterization of silicalite-1 sample

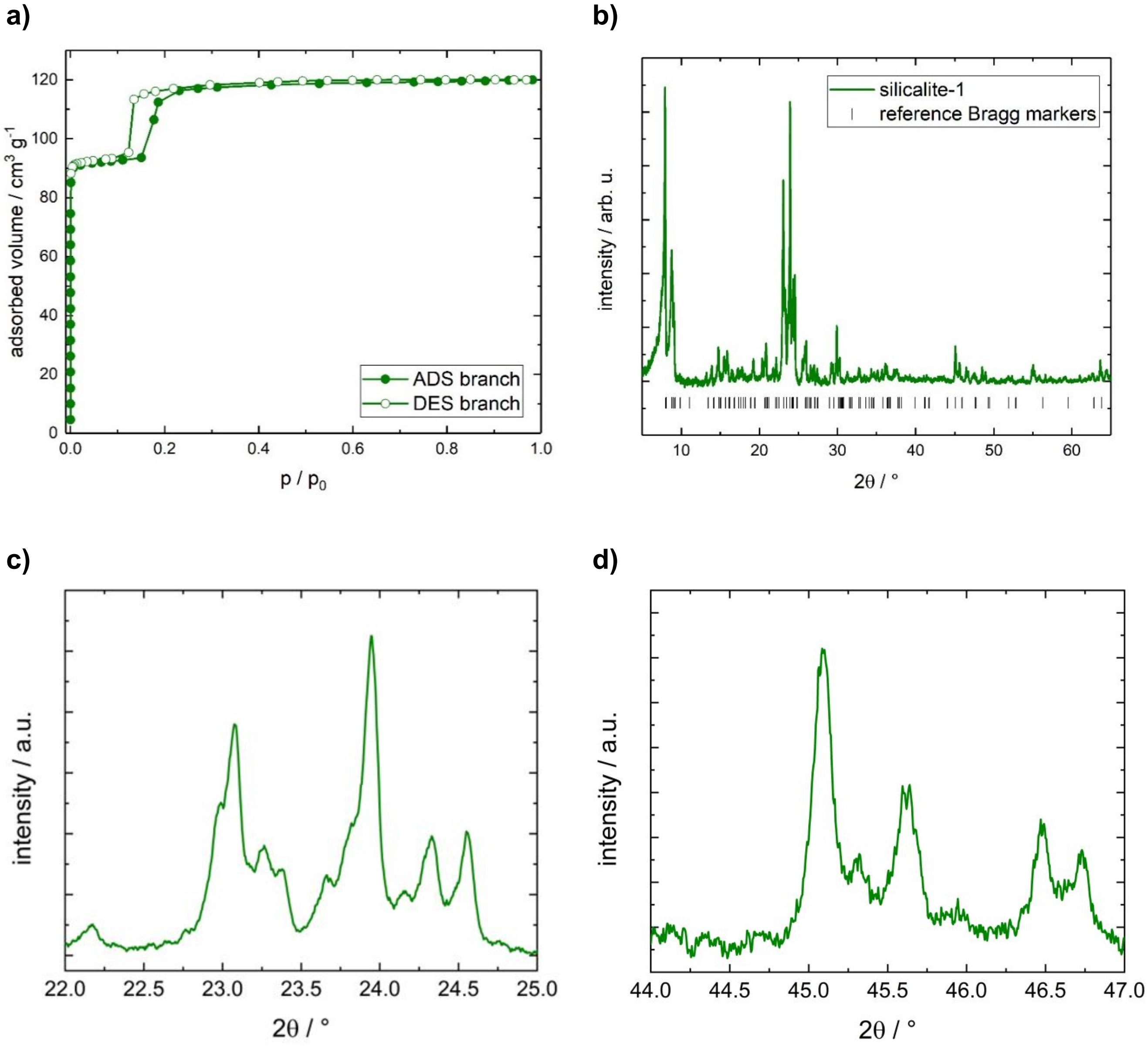

Figure S1. a) Nitrogen adsorption isotherm of silicalite-1 crystals with the adsorption branch indicated by (●) and desorption branch indicated by (○). **b)** X-ray powder diffraction pattern of calcined silicalite-1 and Bragg markers of the reference XRD for MFI framework from the IZA Database of Zeolite Structures. The intervals of **c)** 22-25° and **d)** 44-47° clearly evidence the peaks for the MONO MFI-structure, which is expected for calcined empty MFI-type crystals at room temperature[1–3].

Based on the synthesis mixture (see experimental methods section of the main paper) formation of silicalite-1 crystals with a high Si/Al ratio is expected. Although no elementary analysis was done, two findings confirm this expectation: (1) absence of noticeable OH-bands in the IR spectra in the range of 3800 – 3600 cm^3 and (2) sharp step in the nitrogen adsorption isotherm in a narrow p/p_0 region[4,5]

in the range between 0.1 and 0.2 p/p_0 (see Figure S1a). Both findings are typical for slicalite-1 materials with Si/Al ratios of >100.[5] In our experience with other measurements of individual MFI-type crystals finding (1) even indicates a ratio $\gg$100.

From the nitrogen adsorption data (Figure S1a) the micropore volume may be estimated by extrapolating the asymptote to the plateau region after the step at 0.2 p/p_0 back to the intersection point at p/p_0 = 0, yielding ca. 117.2 cm^3 STP/g. This value is in a range typically found in experimental studies of MFI-type materials.[4,6] Assuming a liquid like density of the adsorbed phase (0.807 g/ml) would yield a micropore volume of ca. 0.18 cm^3/g. However, the isotherm at around 0.2 p/p_0 is associated with a fluid-to-crystalline like phase transition of the adsorbed phase, resulting in a density increase of about 30%.[4] Taking this density change into account, the estimated value of the micropore volume reduces to 0.14 cm^3/g. This value is also obtained when extrapolating back the first plateau to the ordinate at p/p_0 = 0 (ca. 91.4 cm^3 STP/g) and implying liquid like density of the adsorbed phase.

In addition, we have calculated the theoretical pore volume for MFI-type structures MONO, PARA and ORTHO as follows. We used an oxygen atomic radius of 1.6 Å (from the force field used in this work, i.e., Dubbeldam 2004[7]). The size used for the Si atoms is zero, given that they are recessed within the framework. The probe-occupiable void fraction and pore volumes were calculated using Zeo++[8] with a probe radius of 1.86 Å (corresponding to the size of a nitrogen molecule in TraPPE force field[9]). The following Zeo++ command was used: *network -r Dubbeldam-MFI_2004.rad -volpo 1.86 1.86 100000 MFI_mono.cif*. The results are summarized in Table S1 and reside in between the above-mentioned experimental values.

Table S1. Calculated probe-occupiable void fraction and pore volume for all MFI phases using Zeo++.

	Void fraction	Micropore volume (cm^3/g)
MFI_MONO	0.291	0.163
MFI_PARA	0.288	0.161
MFI_ORTHO	0.296	0.165

In accordance with literature, our XRD data clearly shows the peaks for the MONO MFI-structure for the calcined empty crystals at room temperature (Figure S1c,d).[1–3] Depending on composition, synthesis temperature and type of the guest molecule, a guest-induced change of the MFI structure may occur. For benzene a change from MONO to ORTHO is reported for low-Al MFI crystals at room temperature for loadings near 4 molec/uc, i.e. when all intersections are occupied. For higher loadings at 6-8 molec/uc another phase transition from ORTHO to PARA is reported.[2,10] For C_2Cl_4 in silicalite-1 at 298 K similar phase transitions were reported at loadings of 4 molec/uc and 7 molec/uc, respectively.[3] There is a variety of other studies reporting on the guest induced phase transition, a recent work even reports in situ STEM data during benzene adsorption.[11]

As for the above-mentioned examples, also 2- and 3-methylpentane prefer the intersections as adsorption sites, resulting in a pronounced step in the adsorption isotherms as these sites get saturated (see Figure S3). When all intersections are occupied at a loading of 4 molec/uc, sites in channels segments are populated too. As the molecules need to “squeeze” into these segments, the phase transition into the ORTHO structures is promoted. We hence believe that similar phase changes might be observed upon sorption of the methylpentanes for loadings above 4 molec/uc but expect a MONO structure below that loading.

As the main part of the reported diffusivities focused on loadings below 4 molec/uc, the MONO MFI-structure was used in the dcTST simulations.

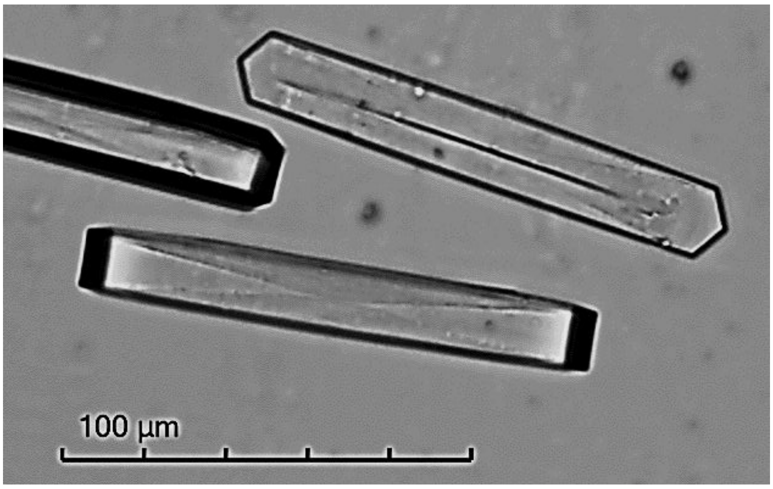

Figure S2. Microscopic image of silicalite-1 indicating the pyramidal segments composing the individual crystal.

Section SI2. IR, simulation, and literature data

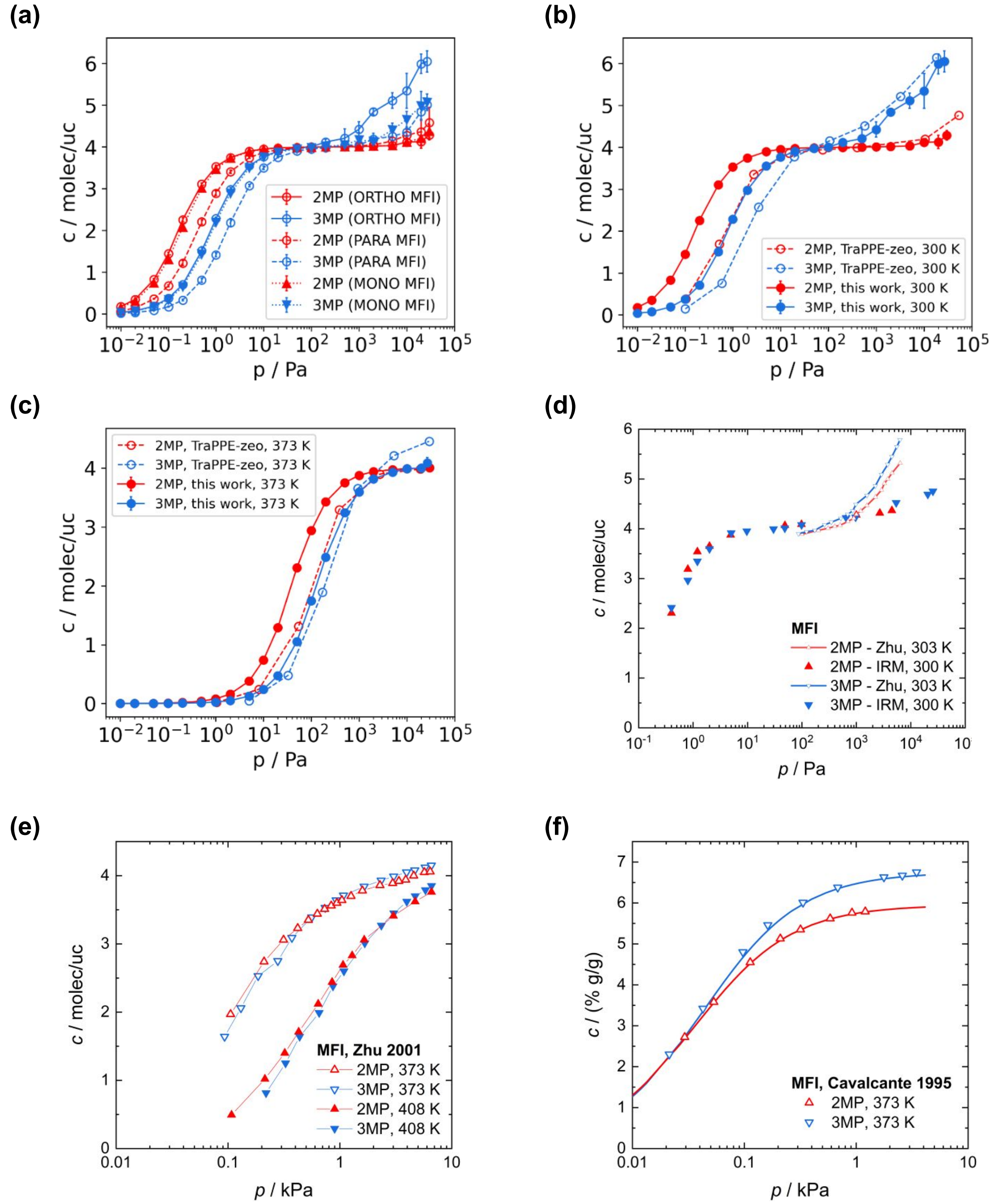

Figure S3. Adsorption isotherms of 2MP and 3MP in MFI-type crystals: **a)** GCMC simulations in ORTHO vs. PARA vs. MONO MFI-structure at 300 K. **b-c)** Comparison of GCMC simulated adsorption isotherms in ORTHO MFI using different force fields: TraPPE-zeo force field[12] and Dubbeldam's force field[7] ("this work") at 300 K and 373 K. **d - f)** IRM measurements vs. literature data from Zhu et al.[13] (d,e) and Cavalcante and Ruthven[14] (f). At room temperature, no experimental data in the low-pressure region were found in literature. At 373 K and 408 K the reported experimental adsorption isotherms of 2MP and 3MP are similar.

The data of Zhu et al. show a tendency for 2MP to be slightly stronger adsorbed than 3MP at low loadings but a reversed order at higher loadings. The same trend was found with IRM at 300 K, albeit the data of Zhu et al. suggest a notably stronger increasing loading for the region > 4 molec/uc. In previous IRM measurements increased molecular interactions were found to cause deviations from the Beer-Lambert law and led to an underestimated concentration by IRM of up to 10-15%. A similar effect may have occurred in this work.

Section SI3. IR micro-imaging data processing

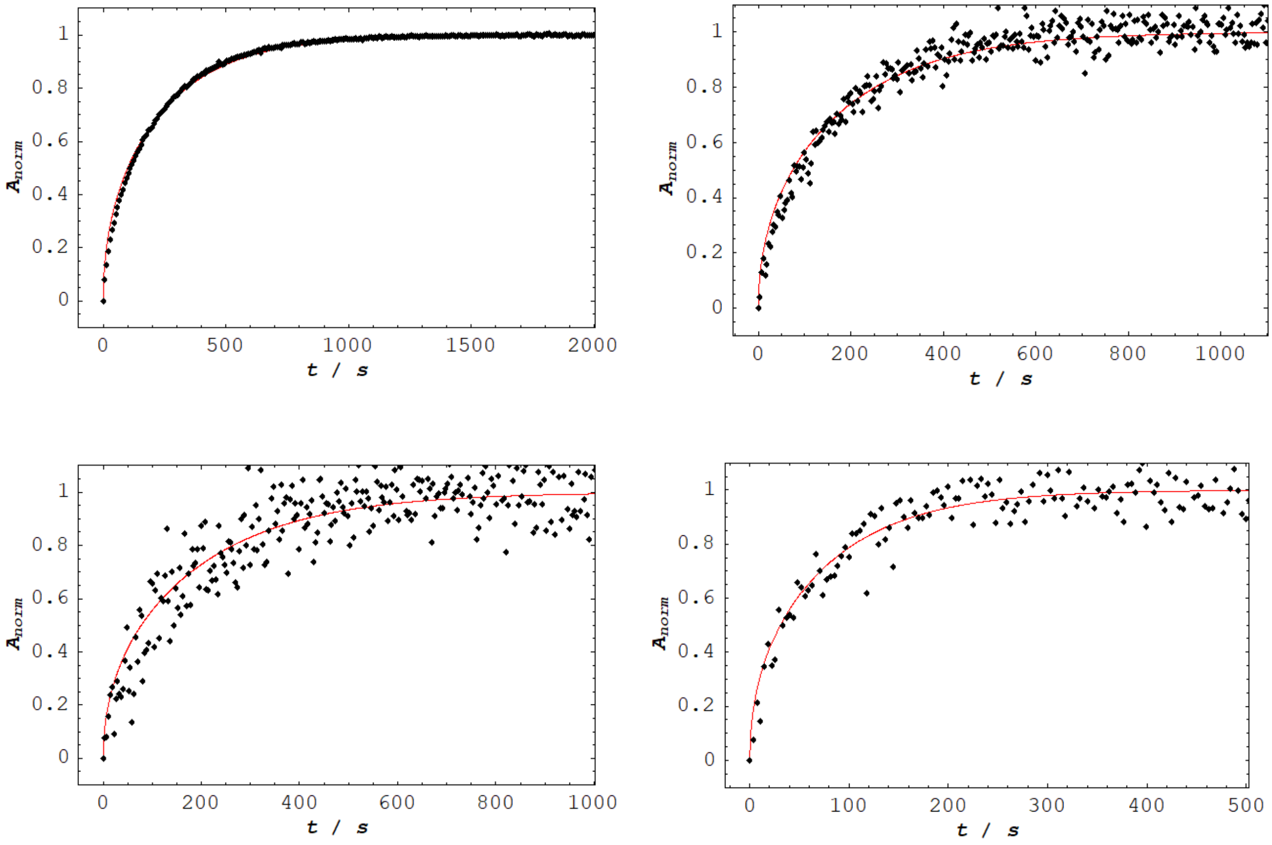

Figure S4. Fits of experimental 2MP uptake curves, which yield experimental D_T values, see Eq. 1 of publication. Examples given for pressure steps 0 – 0.4 Pa, 2 – 5 Pa, 5 – 10 Pa and 100 – 1000 Pa.

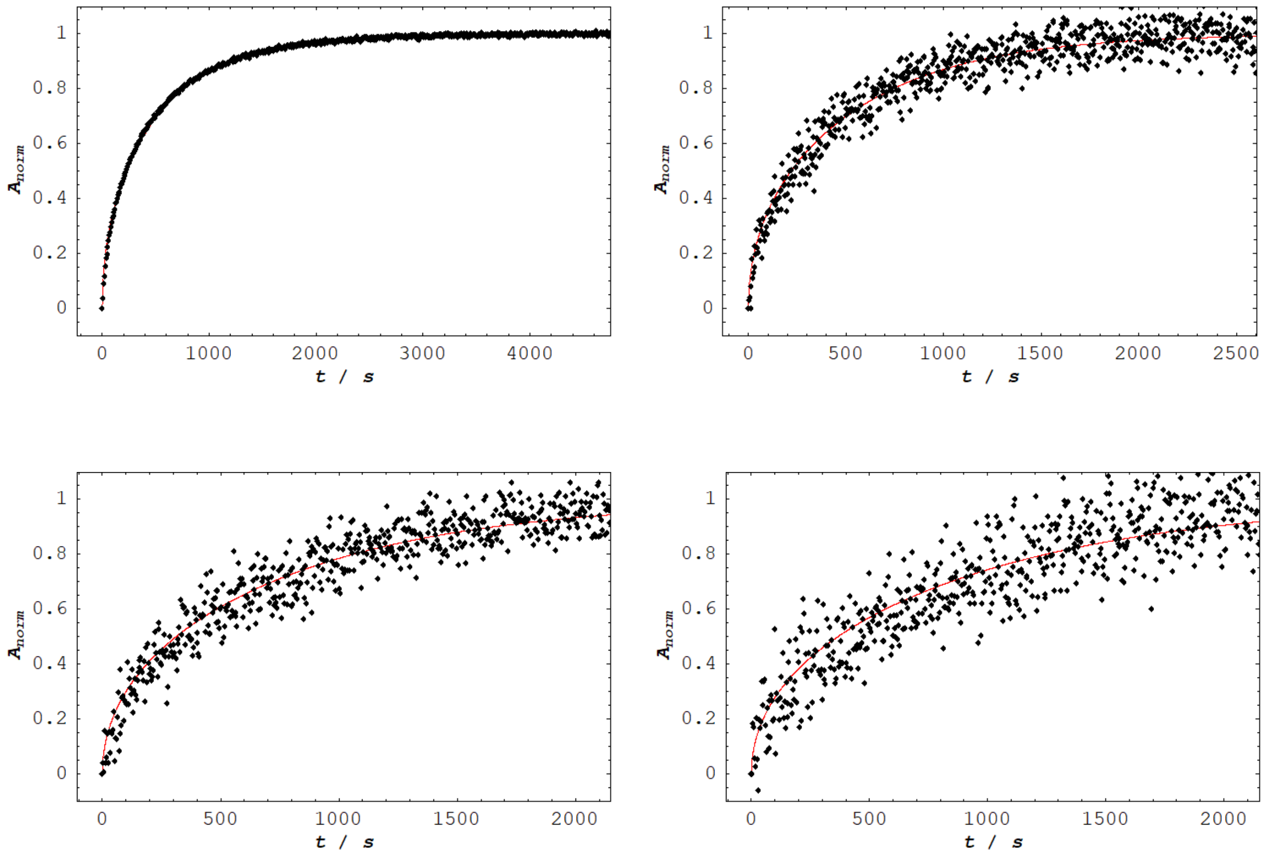

Figure S5. Fits of experimental 3MP uptake curves, which yield experimental D_T values, see Eq. 1 of publication. Examples given for pressure steps 0 – 0.4 Pa, 1.2 – 2 Pa, 2 – 5 Pa and 100 – 1000 Pa.

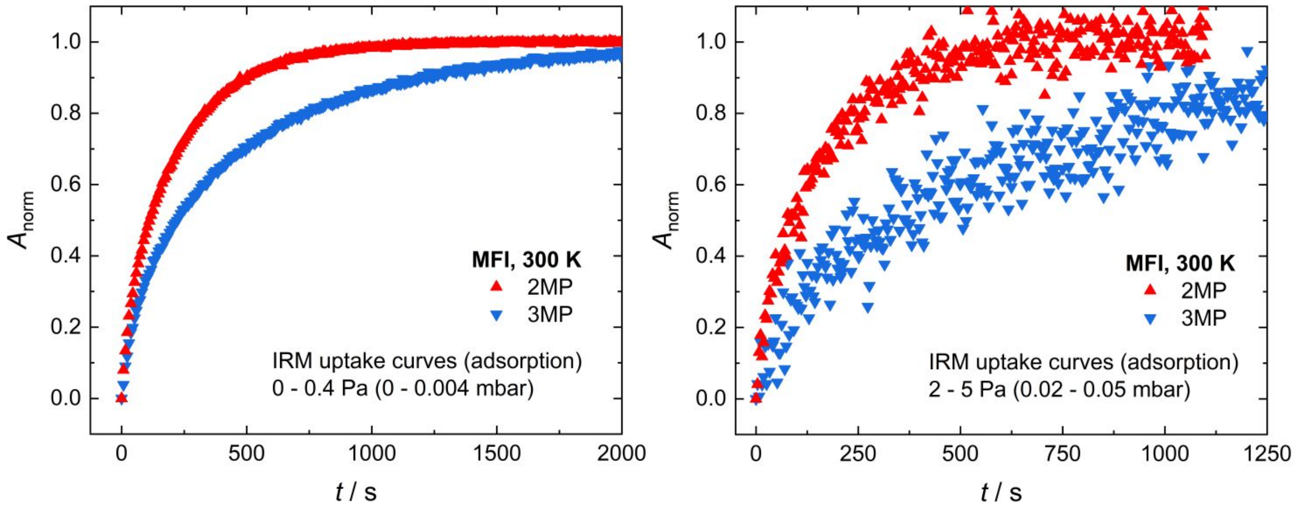

Figure S6. Examples – IRM uptake curves of 2MP vs 3MP: 0 – 0.4 Pa and 2 – 5 Pa.

Section SI4. Details for grand canonical Monte Carlo simulations and transition state theory calculations

Table S2. Non-bonded Lennard-Jones potential parameters for 2MP and 3MP interacting with MFI. 12-6 Lennard-Jones potential is $\upsilon(r) = 4\varepsilon\left[(\sigma/r)^{12} - (\sigma/r)^{6}\right]$, where r is the distance between any two beads. Because the large oxygen atoms shield the much smaller silicon atoms, only interactions between the adsorbate and the oxygen atoms were considered. Interactions between framework atoms were neglected.

Interaction type	ε/k_B [K]	σ [Å]
CH_3-CH_3	108.00	3.76
CH_3-CH_2	77.77	3.86
CH_3-CH	42.85	4.22
CH_3-O	93.00	3.48
CH_2-CH_2	56.00	3.96
CH_2-CH	30.85	4.32
CH_2-O	60.50	3.58
CH-CH	17.00	4.67
CH-O	40.00	3.92

Table S3. Potential parameters for dihedral in alkane models. Intramolecular 1-5 non-bonded interactions should be included.

Type	η_0/k_B [K]	η_1/k_B [K]	η_2/k_B [K]	η_3/k_B [K]	η_4/k_B [K]	η_5/k_B [K]	Potential form
CH_x-CH_2-CH-CH_y	1367.086	4360.147	416.005	-6499.427	-832.004	1646.129	$\cdot_{sion} = \sum_{n=0}^{5} \eta_n \cos^n \phi_{ijkl}$
CH_x-CH_2-CH_2-CH_y	1204.654	1947.740	-357.845	-1944.666	715.690	-1565.572	

Table S4. Potential parameters for bond-stretching in alkane models.

Type	k_s/k_B [K/Å^2]	r_0 [Å]	Potential form
CH_x-CH_x	96500	1.54	$\upsilon_{stretch} = \frac{1}{2} k_s (r - r_0)^2$

Table S5. Potential parameters for bending in alkane models.

Type	k_b/k_B [K/rad^2]	θ_0 [degree]	Potential form
CH_x-CH_x-CH_x	62500	114	$\upsilon_{bend} = \frac{1}{2} k_b (\cos\theta - \cos\theta_0)^2$

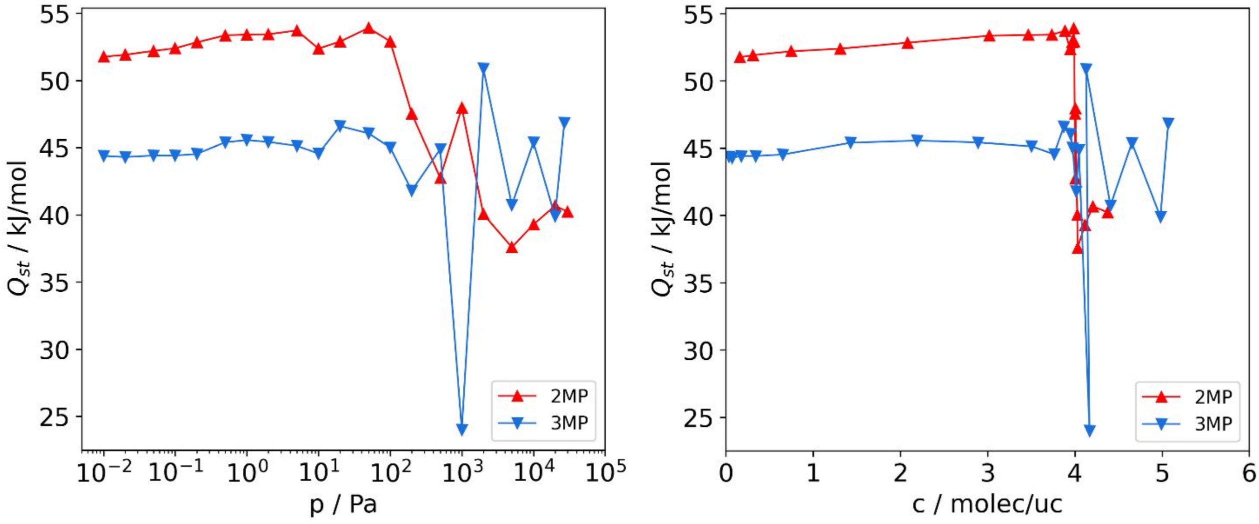

Figure S7. Simulated isosteric heat of single-component 2MP and 3MP adsorption in MONO MFI at 300 K. Left: Isosteric heat versus pressure. Right: Isosteric heat versus adsorption amount.

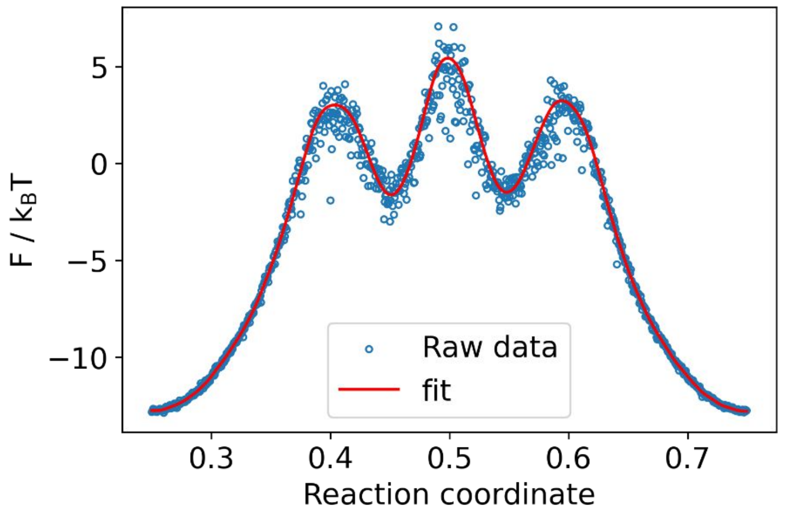

Figure S8. Free energy profile of 2MP along the reaction coordinate for the straight channel in MONO MFI at 300 K. The raw free energy data were fitted to a cubic spline before carrying out the TST integration.

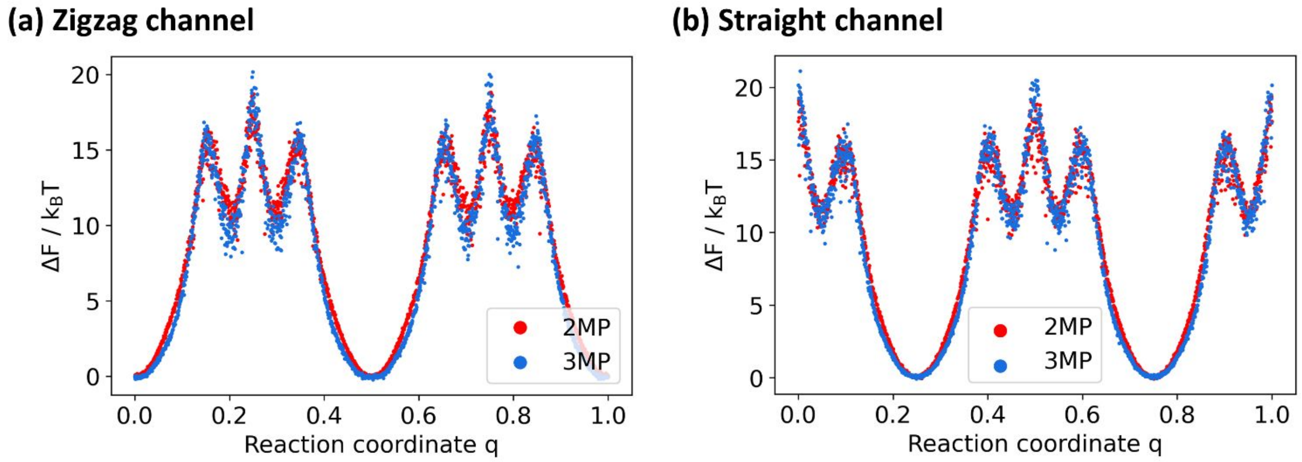

Figure S9. Original free energy data for 2MP and 3MP along the reaction coordinate in (a) zigzag and (b) straight channels in MONO MFI at 300 K. An equivalent, cubic-spline-fitted version is available in Figure 4.

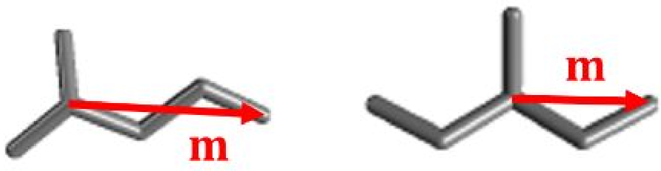

Figure S10. Definition of the molecular tail vector $\mathbf{m}$ for 2MP on the left and 3MP on the right.

The transmission coefficient is defined as[15]

$$\kappa(t) = \frac{\langle \dot{q}(0)\delta(q(0) - q^*)\theta(q(t) - q^*)\rangle}{0.5\langle|\dot{q}(0)|\rangle} \quad \text{(S1)}$$

where $q(t)$ is the reaction coordinate of the bead at time t; $\dot{q}(0)$ is the initial velocity; $\delta(\ldots)$ is the Delta function, and $\theta(\ldots)$ is the Heaviside step function. Eq. (S1) can be rewritten as the summation of the transmission coefficient for trajectories with initial velocity pointing forward, $\kappa_{for}(t)$, and the transmission coefficient for trajectories with initial velocity pointing backward, $\kappa_{bac}(t)$:

$$\kappa(t) = \frac{\langle \dot{q}(0)\delta(q(0) - q^*)\theta(q(t) - q^*)\rangle_{forward}}{0.5\langle|\dot{q}(0)|\rangle} + \frac{\langle \dot{q}(0)\delta(q(0) - q^*)\theta(q(t) - q^*)\rangle_{backward}}{0.5\langle|\dot{q}(0)|\rangle} = \kappa_{for}(t) + \kappa_{bac}(t) \quad \text{(S2)}$$

It is clear from Eq. (S2) that $\kappa_{for}(t) \neq \kappa_{bac}(t)$. In the limit of ideal transmission (i.e., all particles cross the barrier and no re-crossing occurs), we have $\kappa_{for}(t) = 1$ and $\kappa_{bac}(t) = 0$, and thus $\kappa(t) = 1$. The Eq. (S2) was also validated by calculating $\kappa_{for}(t)$ and $\kappa_{bac}(t)$ separately in a modified RASPA2 package, as shown in Figure S11 below (for example of 2MP at 300 K crossing the barrier from 1→2 in the a-direction, corresponding to Figure 4a in the manuscript):

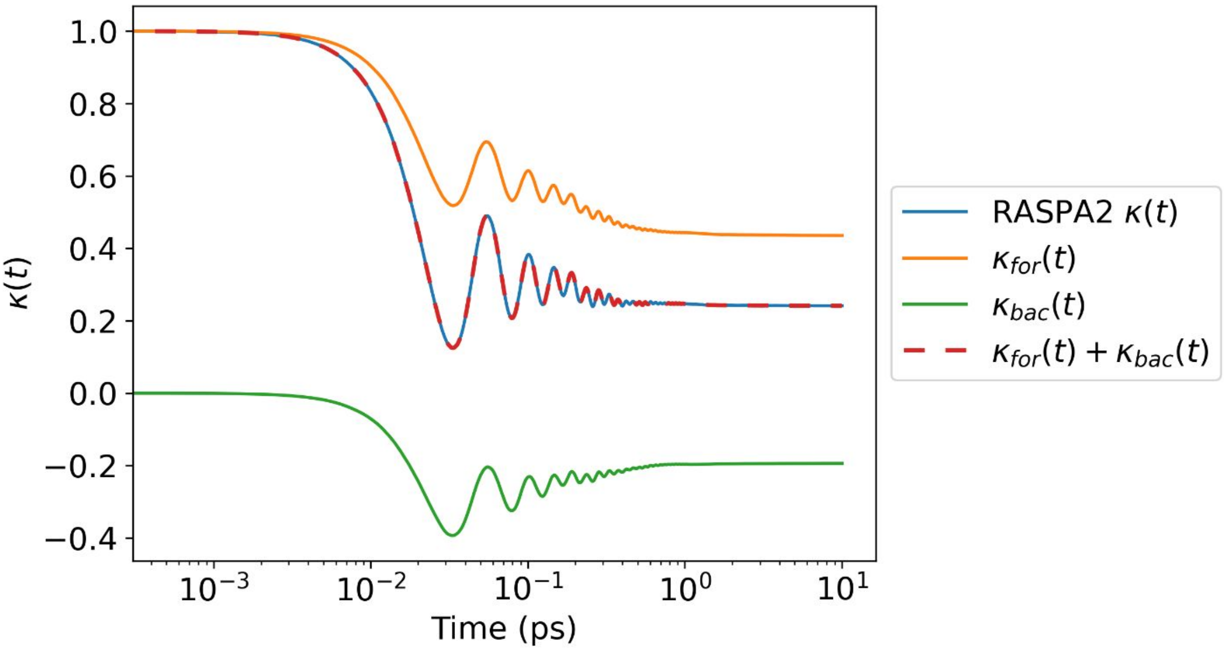

Figure S11. A validation of forward and backward transmission coefficient, in the example of 2MP at 300 K crossing the barrier from 1→2 in the a-direction, corresponding to Figure 4a in the manuscript. The agreement between $\kappa_{for} + \kappa_{bac}$ (dashed red line) and the output from the original RASPA2 software (blue line) validates the Eq. S2 and the implementation in the RASPA2 software.

In this work, we selected the dividing surface to be at the top of the energy barrier. To show that the dynamically corrected hopping rate is independent of the chosen dividing surface, we have performed

an additional dcTST calculation for 2MP at $q^* = 0.14$, a dividing surface that is slightly shifted towards the state 1 in Figure 4a. Comparing to the results when the dividing surface was chosen to be at the top of the barrier ($q^* = 0.15$), we observe a larger TST hopping rate for $q^* = 0.14$ as expected due to the lower energy barrier, as shown in Table S6. Additionally, the corresponding transmission coefficient is smaller, leading to consistent results for the dynamically corrected hopping rate k_{12} in both cases, within uncertainties.

Table S6. Comparison of TST hopping rate, transmission coefficients, and dynamically corrected hopping rates calculated at two different dividing surfaces. Number in the bracket denotes the standard deviation in the last digit.

Dividing surface	k_{12}^{TST} [1/s]	κ_{12}	k_{12} [1/s]
$q^* = 0.15$	3.09741×10^5	0.25241(9)	7.818(3) ×10^4
$q^* = 0.14$	9.97934 ×10^5	0.07842(4)	7.826(4)×10^4

References

(1) van Koningsveld, H.; Tuinstra, F.; van Bekkum, H.; Jansen, J. C. The location of p -xylene in a single crystal of zeolite H-ZSM-5 with a new, sorbate-induced, orthorhombic framework symmetry. *Acta Crystallogr. B Struct. Sci.* **1989**, *45* (4), 423–431. DOI: 10.1107/S0108768189004519.

(2) Mentzen, B.; Lefebvre, F. Flexibility of the MFI Silicalite Framework upon Benzene Adsorption at Higher Pore-Fillings: A Study by X-ray Powder Diffraction, NMR and Molecular Mechanics. *Mat. Res. Bull.* **1997**, *32* (7), 813–820. DOI: 10.1016/S0025-5408(97)00055-X.

(3) Floquet, N.; Coulomb, J. P.; Weber, G.; Bertrand, O.; Bellat, J. P. Structural Signatures of Type IV Isotherm Steps: Sorption of Trichloroethene, Tetrachloroethene, and Benzene in Silicalite-I. *J. Phys. Chem. B* **2003**, *107* (3), 685–693. DOI: 10.1021/jp025701s.

(4) Groen, J. C.; Peffer, L.; Pérez-Ramírez, J. Pore size determination in modified micro- and mesoporous materials. Pitfalls and limitations in gas adsorption data analysis. *Microporous Mesoporous Mater.* **2003**, *60*, 1–17. DOI: 10.1016/S1387-1811(03)00339-1.

(5) Groen, J. C.; Pérez-Ramírez, J. Critical appraisal of mesopore characterization by adsorption analysis. *Appl. Catal. A Gen.* **2004**, *268*, 121–125. DOI: 10.1016/j.apcata.2004.03.031.

(6) Schmidt, W.; Wilczok, U.; Weidenthaler, C.; Medenbach, O.; Goddard, R.; Buth, G.; Cepak, A. Preparation and morphology of pyramidal MFI single-crystal segments. *J. Phys. Chem. B* **2007**, *111* (48), 13538–13543. DOI: 10.1021/jp075934p. Published Online: Nov. 8, 2007.

(7) Dubbeldam, D.; Calero, S.; Vlugt, T. J. H.; Krishna, R.; Maesen, T. L. M.; Smit, B. United Atom Force Field for Alkanes in Nanoporous Materials. *J. Phys. Chem. B* **2004**, *108* (33), 12301–12313. DOI: 10.1021/jp0376727.
(8) Willems, T. F.; Rycroft, C. H.; Kazi, M.; Meza, J. C.; Haranczyk, M. Algorithms and tools for high-throughput geometry-based analysis of crystalline porous materials. *Microporous Mesoporous Mater.* **2012**, *149* (1), 134–141. DOI: 10.1016/j.micromeso.2011.08.020.
(9) Bae, Y.-S.; Yazaydin, A. O.; Snurr, R. Q. Evaluation of the BET method for determining surface areas of MOFs and zeolites that contain ultra-micropores. *Langmuir* **2010**, *26* (8), 5475–5483. DOI: 10.1021/la100449z.
(10) Pera-Titus, M. Thermodynamic Analysis of Type VI Adsorption Isotherms in MFI Zeolites. *J. Phys. Chem. C* **2011**, *115* (8), 3346–3357. DOI: 10.1021/jp109449q.
(11) Xiong, H.; Liu, Z.; Chen, X.; Wang, H.; Qian, W.; Zhang, C.; Zheng, A.; Wei, F. In situ imaging of the sorption-induced subcell topological flexibility of a rigid zeolite framework. *Science (New York, N.Y.)* **2022**, *376* (6592), 491–496. DOI: 10.1126/science.abn7667. Published Online: Apr. 28, 2022.
(12) Bai, P.; Tsapatsis, M.; Siepmann, J. I. TraPPE-zeo: Transferable Potentials for Phase Equilibria Force Field for All-Silica Zeolites. *J. Phys. Chem. C* **2013**, *117* (46), 24375–24387. DOI: 10.1021/jp4074224.
(13) Zhu, W.; Kapteijn, F.; van der Linden, B.; Moulijn, J. A. Equilibrium adsorption of linear and branched C6 alkanes on silicalite-1 studied by the tapered element oscillating microbalance. *Phys. Chem. Chem. Phys.* **2001**, *3* (9), 1755–1761. DOI: 10.1039/b100941i.
(14) Cavalcante, C. L., JR.; Ruthven, D. M. Adsorption of Branched and Cyclic Paraffins in Silicalite. 1. Equilibrium. *Ind. Eng. Chem. Res.* **1995**, *34* (1), 177–184. DOI: 10.1021/ie00040a017.
(15) Frenkel, D.; Smit, B. *Understanding Molecular Simulation*; Academic Press, 2023. DOI: 10.1016/C2009-0-63921-0.
